# IGF-I combined with exercise improve diabetes-induced vascular dysfunction in heart of male Wistar rats

**DOI:** 10.34172/jcvtr.2021.54

**Published:** 2021-12-16

**Authors:** Zahra Niknam, Mahrokh Samadi, Ataollah Ghalibafsabbaghi, leila Chodari

**Affiliations:** ^1^Proteomics Research Center, Shahid Beheshti University of Medical Science, Tehran, Iran; ^2^Nephrology and Kidney Transplant Research Center, Clinical Research Institute, Urmia University of Medical Sciences, Urmia, Iran; ^3^Student Research Committee, Urmia University of Medical Sciences, Urmia, Iran; ^4^Neurophysiology Research Center, Cellular and Molecular Medicine Institute, Urmia University of Medical Sciences, Urmia, Iran; ^5^Department of Physiology, Faculty of Medicine, Urmia University of Medical Sciences, Urmia, Iran

**Keywords:** Diabetes, Angiogenesis, Heart, VEGF-A, TSP-1, NF-кβ

## Abstract

***Introduction:*** This research investigates the impact of insulin-like growth factor-I (IGF -I)and exercise on mediators associated with angiogenesis (VEGF-A, TSP-1, and NF-кβ) and capillarization status of the diabetic rats’ hearts.

***Methods:*** Splitting of forty Wistar male rats into five groups occurred as following: control,diabetes, diabetes+IGF-I, diabetes+exercise, and diabetes+exercise+IGF-I.Through intraperitoneal administration of 60 mg/kg streptozotocin, the condition of Type 1diabetes was escalated. After four weeks of treatment with IGF-I (2 mg/kg/day) or treadmill exercise (17 m/min, zero degrees slope, 30 min/day), in the heart, microvascular density and protein levels of VEGF-A, TSP-1, and NF-кβ were determined by H&E staining and ELISA,respectively.

***Results:*** Within the diabetic group, observations present a significant decrease in VEGF-A and MVD levels, whereas an increase in the TSP-1 and NF-Κb levels. While these impacts were reversed by either IGF-I or exercise treatments, simultaneous treatment had synergistic effects. Moreover, among diabetic rats, undesirable histologic alterations of the heart were demonstrated, including myonecrosis, interstitial edema, hemorrhage, and mononuclear immune cell infiltration, whereas treatments improved these changes.

***Conclusion:*** These data manifest that IGF-I and exercise can increase the cardiac angiogenesis of diabetic rats through increasing expression of VEGF-A, and decreasing TSP-1 and NF-кβproteins level, also can improve myocardial tissue damages.

## Introduction

Diabetes is a major leading cause of death worldwide, and it incurs substantially to healthcare costs. The global diabetes prevalence is estimated to enhance drastically in the future decades due to the world’s older population growth, on the other hand, with the increasing burden of overweight and obesity. In 2017 diabetes counted for 425 million cases, and its prevalence will increase to 629 million by 2040.^1^ Among risk factors of cardiovascular diseases (coronary heart disease, stroke, peripheral arterial disease, congestive heart failure, and cardiomyopathy), a major one is allocated to diabetes mellitus.^2^ Within diabetic patients, stated cardiovascular diseases play a crucial role in mortality and morbidity.^2^

A presumable reason to explain the soaring risk for cardiovascular diseases in diabetic patients is the unbalanced angiogenic stimulator and inhibitor factors.^3^ As a normal and vital physiological process, angiogenesis is the blood vessel formation from the present vasculature. It is settled on maintaining the balance between angiogenic factors (angiopoietins, FGF2, TGF-β, and VEGF) and angiostatic factors (endostatin, TSP-1, and angiostatin).^4^ Microvascular insufficiency in diabetes mellitus plays a principal role in the development of cardiomyopathy. The data collected show that a gradual decrease in microvascular and impaired angiogenesis response has occurred in chronic ischemia and alongside the diabetes’ progresssion. These microvascular complications in diabetic patients may cause decreased perfusion of the myocardium and lead to cardiovascular diseases.^5^ Therefore, this study investigated molecular mediators associated with the angiogenesis process in the heart of diabetic rats.

As a crucial angiogenic factor, Vascular endothelial growth factor (VEGF), reportedly is related to many pathological complications, such as diabetes mellitus and cardiovascular disorders (including ischemic heart disease, coronary artery disease, atherosclerosis, and strokes).^2,6^ It has been reported that the reduction of VEGF level in diabetic cardiac tissue has a possible role in the impairment of myocardial angiogenesis and leads to impaired collateral formation. It presumably has a considerable role in soaring of morbidity and mortality risks among diabetic patients.^5^ Therefore, the normalization of the down-regulated cardiac expression levels of VEGF is proposed to improve diabetic myocardial complications.^7^

Thrombospondin-1 (TSP)-1, a potent anti-angiogenic mediator and a large extracellular matrix-associated protein, is upregulated in the diabetic cardiovascular system. TSP-1 induces endothelial cell apoptosis by activating CD36/p59fyn/p38 Mitogen-Activate Protein Kinase (MAPK) pathway. Within the diabetic myocardium, assumably, TSP-1 has a role in undermining angiogenic signals, inducing vascular rarefaction and remodeling the heart.^8,9^

Nuclear factor-кβ (NF-кβ) is another molecular mediator that plays an important role in the vascular complications’ pathogenesis of diabetes.^10^ Persistent hyperglycemia activates NF-кβ that is a common mechanism of angiostatic agents, resulting in angiogenesis inhibition^11^ and induction of diabetic cardiomyopathy.^12^

The potent effects of insulin-like growth factor-I (IGF-I) on proliferation and angiogenesis in different target tissues are identified.^13^ On the other hand, various studies have reported a decline in serum IGF-1 levels in diabetic animals or patients; therefore, it is an attractive therapeutic candidate for diabetes.^14^ IGF-1, as a potent growth hormone, is crucial to induce essential functions of VEGF. Furthermore, when used together, IGF-1 and VEGF demonstrated complementary therapeutic effects in post-infarction heart failure.^15^ A recent study expresses the TSP-1 suppression by IGF-I in primary cultures of porcine granulosa cells, but its mechanism has not been investigated.^16^

Improvement in skeletal muscle capillarization and rise in VEGF expression by exercise training is a well-known phenomenon in healthy humans and animals.^3^ Also, recent studies showed that exercise training exerts beneficial effects on the expression of pro-angiogenic factors in diabetic cardiac and capillaries.^2,3^

In light of the above findings, to our knowledge, either sole IGF-I effects or its collaboration with exercise on protein alterations of NF-кβ, VEGF-A, and TSP-1 have not been investigated in the heart tissue of a diabetic model. So, the present work aims to assess the IGF-I and exercise’s impression on protein expressions of NF-кβ, VEGF, and TSP-1 alongside the heart tissue microvascular density modifications of STZ-diabetic rats.

## Materials and Methods

### Animals and study design

In this study, 40 male Wistar rats weighing 250-300 g were randomly divided into 5 groups (8 rats in each group):

1. Control group (Cont): In this group, healthy rats were kept for 4 weeks without any intervention.
2. Diabetic group (Dia): In this group, rats became diabetic and were kept for 4 weeks.
3. Diabetes + IGF-I group (Dia+IGF-I): In this group, diabetic rats received daily IGF-I (2mg/kg/rat) for 4 weeks.
4. Diabetes + exercise group (Dia+E) : In this group, dibetic rats exercised for 4 weeks.
5. Diabetes + IGF-I + exercise group (Dia+IGF-I+E): In this group, the diabetic rats exercised and received IGF-I for 4 weeks.

### Induction of diabetes

To induce diabetes, rats were injected intraperitoneally with 60 mg/kg streptozotocin (Sigma, St. Louis, Missouri, USA). Streptozotocin was dissolved in 10mM sodium citrate with 0.9% sodium chloride at pH = 4.5. According to this method, type 1 diabetes generally develops in rats 72 hours after injection. To diagnose and confirm diabetes, by causing a slight injury via Lancet in the rat tail, a drop of blood was placed on a glucometer strip and read by a glucometer (Boehringer Mannheim Indianapolis, IN). The blood glucose level was ≥ 300 mg/dL (16.67 mmol/L), which is a diabetes indicator.^17^

### IGF-I treatment

IGF-I (Amino Acids, P.F, Tianjin, China) was injected 2 mg/rat/day subcutaneously six days a week for four weeks.^17^ All injections occurred between 8 and 9 in the morning. Other groups, absence of IGF-I treatment, were administered 0.2 ml/rat normal saline.

### Exercise protocol

For exercise, a rat treadmill machine was used. Rats were forced to run daily (6 days a week) for 30 minutes at a 17 m/min speed with a zero-degree slope for four weeks. To minimize treadmil running physical stress and adaptation, the animals exercised for 10 minutes on the first day, and in the following days, 5 minutes per day was added to the exercise time until it finally reached 30 minutes per day. (First day: 10 minutes, second day 15 minutes, third day 20 minutes, fourth day 25 minutes, fifth day to the end of a month 30 minutes). The exercise took place in the mornings between 9 – 12 AM. Rats that were not treated with exercise were also taken to the exercise room and left on a treadmill for 30 minutes.^18^

### Tissue processing and protein measurement by ElSA method

Animals were anesthetized with ketamine (80 mg/kg) and Xylazine (5 mg/kg) at the end of the experiment. Heart tissues were rapidly excised, washed with saline 0.9%, and stored immediately at –80°C for further analysis. The left ventricle tissue samples were accustomed to level measure VEGF (Cat. No：ZB-10659S-R9648), TSP-1 (Cat. No：ZB-10687S-R9648), and phosphorylated and activated form of NF-Ƙb (Cat. No：ZB-10287-R9648) by high-sensitivity ELISA kits (Zelbio, Germany). To preserve proteins from damage, a portion of the tissue samples was homogenized in 1ml potassium phosphate buffer (pH 7.2 to 7.4) containing an anti-protease cocktail. Then, the homogenate was centrifuged for 20 min at 4°C at 1000 × g. In the end, the outcome supernatant was cleared, and target proteins were extracted for ELISA analysis.

### Histological evaluation

Succeding to exercise and IGF-I therapy, and upon the rats’ scarifications, samples from the left ventricle heart were derived and prepared for the upcoming examinations. Samples of this organ were placed in a 4% paraformaldehyde solution and subsequently sectioned into 5-μm sections transversely. Sections were stained with hematoxylin and eosin to observe histologic changes with a magnification of ×20 (Olympus, USA). The severity of the injury was specified as cardiomyocyte injury by the predetermined scoring system (Solez et al 1979). In which the severity of interstitial edema, cell lysis, Mononuclear infiltration cell, and necrosis was scored as following: 0, < 25 cell count (no reaction); 1, > 25 cell count (mild reaction); 2, > 50 cell count (moderate reaction); and 3, > 100 cell count (severe reaction). A blinded expert performed the histological analysis.

The number of microvesselswas accustomed to evaluating the left ventricle angiogenesis level, and the microvasculature density was measured as angiogenesis index. For this purpose, five divisions with 50mm spans were derived from a vivid vasculature to be examined under a light microscope by two blind (to experiment’s situation) observers. So, calculations of each microvessels’ number presented every section’s average microvessels’ quantity.^19^

### Statistical analysis

Data distribution was controlled using the Kolmogorov–Smirnov test. The data were normally distributed and analyzed using parametric techniques. Regarding quantitive data comparison, Turkey’s test and One-way analysis of variance (ANOVA) were chosen. And, Mann- Whitney U was looked upon for histologic changes’ testing. Statistical analysis of data was carried out using SPSS statistic software (Version 17.0). Also, statistical significance was defined as *P* < 0.05.

## Results

### Effects of exercise and IGF-I on protein levels (VEGF-A,TSP-1, and p-NF-Ƙb) in the heart tissue

**Figure 1:**
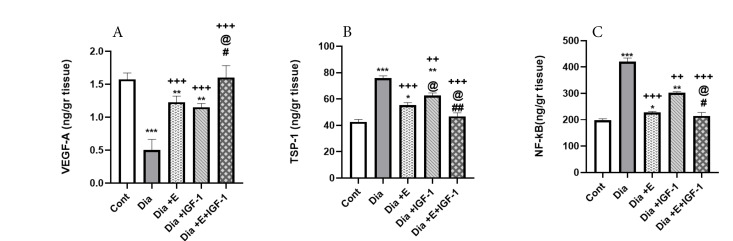
Effect of IGF-I and exercise on VEGF-A (A), TSP-1 (B )and NF-κB (C) expression in heart tissue of diabetesgroups. Values are expressed as Mean ± SD.^**^P < 0.01, ^***^P < 0.001 vs Cont group. ^++^*P* < 0.01, ^+++^*P* < 0.001vs Dia group. ^@^*P* < 0.05 vs Dia+Exe group. #*P* < 0.05, ##*P* < 0.01vs Dia+IGF-I group

The ELISA assay outcome exhibited a substantial drop (compared to the control group) in the heart tissue VEGF-A protein levels among the diabetic group (*P* < 0.001) (Figure 1A). In contrast to the diabetic group, the four-week exercise or IGF-I treatment of the diabetic rats soared (*P* < 0.001) VEGF-A protein levels (illustrated in Figure 1A). However, it was still below the control group. Also, the integrated IGF-I and exercise therapy surged the VEGF-A protein levels regarding the diabetic (*P* < 0.001), Dia+E (*P* < 0.05), and Dia+IGF-I (*P* < 0.05) groups. Moreover, combination therapy was able to increase the VEGF-A levels very close to that in the control group.

Also, amounts of the NF-кβ and TSP-1 proteins (Figures 1B and Figure 1C) were significantly (*P* < 0.001) increased in the heart tissue of the diabetic group compared with the control group. But these protein levels still are higher than the control group. In comparison with the diabetic group and as shown in Figures 1B and 1C, exercise (*P* < 0.001) and IGF-I (*P* < 0.01) treatments of the diabetic rats significantly decreased the amounts of the NF-кβ and TSP-1 proteins. Moreover, mixed IGF-I and exercise therapy for four weeks plunged the amounts of the NF-кβ and TSP-1 proteins comparing with the Dia+E (*P* < 0.05) and the diabetic (*P* < 0.001) groups. Also, r­egarding the Dia+IGF-I group, a drastic (*P* < 0.01) level decline in TSP-1 proteins was recorded in the Dia+E+IGF-I group. According to the results, exercise and IGF-I were able to show a synergistic effect in reducing TSP-1 protein.

### Effects of exercise and IGF-I on myocardial tissues in type I diabetic rats

**Figure 2:**
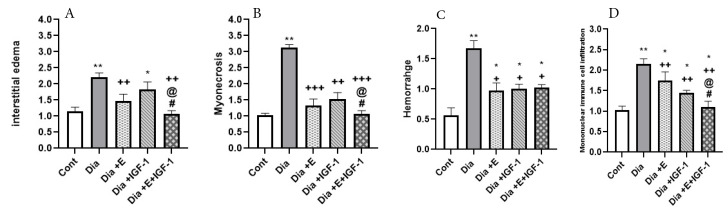
Effect of IGF-I and exercise on Edema (A), Necrosis (B), Hemorrhage (C), and Mononuclear infiltration cell (D), in heart tissue in the diabetes groups. Data are expressed as mean ± SEM for 8 animals. * *P* < 0.05, ** *P* < 0.01 vs the Cont group. +*P* < 0.05, ++ *P* < 0.01, +++ *P* < 0.001 vs the Dia group. @ *P* < 0.05 vs the Dia+E group. # *P* < 0.05, vs the Dia+IGF-I group. E: Edema, N:Necrosis, H:Hemorrhage, MNIC: Mononuclear infiltration cell

As Figure 2, Mann-Whitney U analyzation present, regarding the control group, there’s a considerable rise (*P* < 0.01) in interstitial edema, mononuclear immune infiltration, and myonecrosis in the Dia group. Also, in the diabetic group, regarding the control group, a shrink in interstitial edema (*P* < 0.01) and leukocyte infiltration (*P* < 0.01) was the outcome of exercise treatment. Furthermore, as shown in Figure 2, the result of following the IGF-I treatment in rats for four weeks was a drastic diminishment in leukocyte infiltration (*P* < 0.01), interstitial edema (*P* < 0.05), myonecrosis (*P* < 0.01), and hemorrhage (*P* < 0.05). Among diabetic rats, integrated IGF-I and exercise treatment demonstrated a considerable enhancing impact on damages of myocardial tissue and prominently diminished interstitial edema (*P* < 0.01), leukocyte infiltration (*P* < 0.01), hemorrhage (*P* < 0.05), and myonecrosis (*P* < 0.001) in comparison to Dia groups. Interestingly, when compared to Dia+ IGF-I and Dia+E groups, interstitial edema, leukocyte infiltration, and myonecrosis considerable diminishment (*P* < 0.05) also resulted from the combined therapy. So, it is cleared that combined therapy showed a synergistic effect on histopathological changes in the heart tissue of diabetic rats.

### Effects of exercise and IGF-I on microvascular density (MVD) in type I diabetic rats

**Figure 3:**
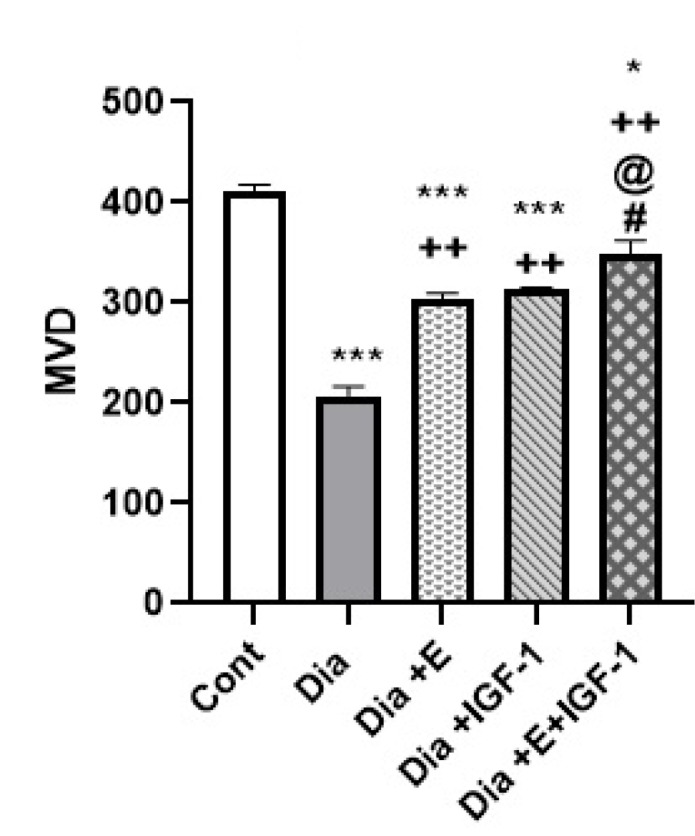
Effect of IGF-I and exercise on MVD of heart tissue in the diabetesgroups. Data are expressed as mean ± SEM for 8 animals. * *P* < 0.05, *** *P* < 0.001 vs the Cont group. ++ *P* < 0.01 vs the Dia group. @ *P* < 0. 05 vs the Dia+E group. # *P* < 0.05 vs the Dia+IGF-I group.

**Figure 4:**
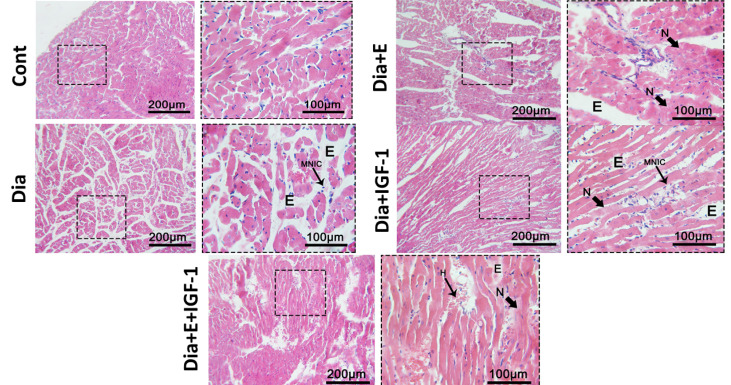
The histological light microscopy images of H&E stained sections in heart of rats. (×40 HE). Cont:control group, Dia:Diabetes group, Dia+E: Diabetes+Exercise group, Dia+ IGF-I:Diabetes+IGF-I group, Dia+E+ IGF-I : Diabetes+Exercise+ IGF-I group

Acording Figure 3 and Figure 4, contrast to the control group, one-way ANOVA revealed a plunge in the heart tissue MVD level in the diabetic group. While treatment of Dia groups with exercise and IGF-I surged (*P* < 0.01) the MVD level regarding the Dia group (Figure 3). According to Figure 3, also four weeks of combination therapy with IGF-I and exercise in diabetic rats significantly enhanced the level of MVD in the heart tissue in comparison with the Dia (*P* < 0.01), Dia+E, and Dia+IGF-I (*P* < 0.05) groups, so it should be mentioned that combination therapy depicted a synergistic effect on MVD.

## Discussion

Diabetes mellitus is the main risk factor for cardiovascular disease, which induces endothelial dysfunction and decreased angiogenesis in the cardiac tissue.^20-22^ In the present study, originally and for the first time, the combined IGF-I and exercise therapy’s impact on microvascular density (MVD), proteins involved in angiogenesis, and histological changes of the STZ-induced with insulin-dependent diabetes mellitus (Type I) rat model’s heart tissue were analyzed. Our results from following uncontrolled four weeks of diabetes demonstrated that protein expressions of MVD and VEGF-A were significantly reduced, and the heart tissue protein levels of NF-кβ and TSP-1 were increased. In addition, obnoxious histologic alterations (interstitial edema, mononuclear immune cell infiltration, hemorrhage, and myonecrosis) were prompted too. The rise of MVD and VEGF-A expression alongside the abate of NF-кβ and TSP-1 expression was also revealed from the four-week diabetic rats’ IGF-I and exercise treatment study. Furthermore, these treatments led to a significant decrease in histopathological damage in the heart of diabetic rats.

Consistent with the results of our study, suppression of angiogenesis in the diabetic heart and skeletal muscle of human and animal models has been observed.^23-25^ but its related pathway is poorly known.^13^

Specific molecular angiogenesis-related mediators, including NF-кβ, VEGF-A, and TSP-1, are considered critical in regulating vessel development.^11,26^ Various studies determine VEGF-A’s regulatory task in cardiac angiogenesis as a strong and essential proangiogenic factor.^6,27^ This study demonstrates a shrink in MVD and VEGF-A protein expression in the heart tissue of diabetic rats. Our results, consistent with previous studies, indicate that diabetes mellitus is associated with decreased cardiac VEGF-A and angiogenic disorders.^2,5^

Previous studies have shown that TSP-1 as a potent angiostatic mediator is markedly upregulated in many organs, including the kidney,^28^ the heart,^8^ and blood vessels^29^ in animal models of diabetes. Also, our findings in this study revealed that TSP-1 protein level in the heart tissue of diabetic rats was significantly higher than in healthy rats. The mechanism underlying increased TSP-1 synthesis is unknown; however, extensive evidence indicates that the TSP-1 manufacture stimulation is underlined by hyperglycemia, which occurs via specific vascular cell pathways that include triggering glucose-mediated hexosamine.^9^

Also, the present study revealed that an increase in TSP-1 protein level in the heart of a diabetic rat is associated with a decrease in VEGF-A protein level and MVD. It is reported that TSP-1 inhibits VEGF signaling by direct binding and prevention of it’s release from the matrix, as well as by inhibition of VEGF receptor phosphorylation.^30^ TSP-1 can also cause endothelial cell apoptosis via a CD36/fyn-mediated pathway.^31^ Third, TSP-1 can directly inhibit endothelial cell cycle progression in a CD36-independent manner or prevent endothelial cell migration through interactions with β1 integrins.^32^ Fourth, TSP-1-induced nitric oxide signaling inhibition may be associated with the angiostatic signaling activation in endothelial cells.^33^ Gonzalez-Quesada et al also demonstrated that TSP-1 overexpression in the heart of a diabetic mice model inhibits chamber dilation by maintaining the matrix of cardiac fibroblasts and induces capillary rarefaction by results that may include angiopoietin-2 upregulation.^8^

High levels of advanced glycation end products in diabetic conditions can increase NF-кβ expression through different mechanisms.^10^ Our findings in this study have also revealed that the amount of NF-кβ protein in diabetic rats’ hearts was significantly higher than in healthy rats. The transcription factor NF-кβ has recently been related to several aspects of angiogenesis. It has been reported that NF-кβ overexpression can activate pro-angiogenesis genes, such as VEGF, IL-8, and MMP-9.^11^ In contrast, many recent studies have associated angiogenesis inhibition with NF-кβ activation.^11^ The outcome of numerous arguments on its dual activity has concluded that stimulus, cell type, and context of activation affect the eventual consequence of NF-кβ activation.^11^ According to our results, in comparison to healthy rats, diabetic rats had a decline in MVD and angiogenesis followed by an increase in the heart tissue proteins of NF-кβ proteins. The presence of NF-кβ binding sites on TSP-1 promoters has been exhibited in previous reports.^34^ Based on our findings, the rise of TSP-1 in diabetic rats can be drawn from a surpass of NF-кβ in hyperglycemia state.

Diabetes-related diseases of cardiac can be followed by a proangiogenic factors reduction in the diabetes cardiac tissue. Therefore, administrating angiogenesis augmentative agents could be of use. According to previous studies, exercise training or IGF-I positively affects on the skeletal muscle vasculature, sciatic nerve, and expression of proangiogenic factors. Therefore, here for the first time, the effects of exercise, IGF-I, and combination therapy on the production of angiogenic and antiangiogenic proteins, as well as capillarization, were studied in the heart tissue of diabetic rats. The results showed that in the groups treated with exercise, VEGF protein and MVD increased, and TSP-1 and NF-кβ proteins decreased compared to the sedentary rats. Indeed, diabetes induction reduces angiogenic factors while increasing antiangiogenic factors. However, exercise training reverses this effect. In agreement with our study, Erekat et al investigated the treadmill exercise effects on the expression of cardiac VEGF proteins in Insulin-dependent diabetes mellitus (type I) rats.^2^ They showed that regarding the sedentary control, among sedentary diabetic rats, VEGF protein expressions of the heart abated. Despite that, in the diabetic rats (in contrast to the sedentary diabetic rats), the VEGF cardiac tissue protein expression surged upon exercise training. In another study, Broderick et al demonstrated that eight weeks of moderately-intense exercise training is related to enhanced expression of key angiogenic markers, potentially through increasing nitric oxide availability in hearts of diabetic mice.^35^ On the other hand, exercise training lowered the production of key cytokines implicated in the evolution of endothelial dysfunction and insulin resistance in cardiac muscle. Moreover, in line with our work, Liu et al demonstrated that moderate exercise training inhibits NF-кβ signaling in muscles of diabetic mice.^36^ Our histological findings also demonstrated the beneficial role of exercise in a clear interstitial edema shrink and infiltration of leukocytes compared to the diabetic group.

It has been shown that IGF-I stimulates angiogenesis and increases blood flow in the diabetic limb, as well as increases VEGF expression in muscle.^13^ Also, compared to the untreated group, this study’s presented data exhibits that IGF-I treatment raises MVD and VEGF levels and lowers diabetic cardiac NF-кβ and TSP-1 expression. McGray et al indicated that IGF-I quickly suppressed TSP-1 protein and mRNA expression in cultured granulosa cells.^37^ It was reported that increased insulin signaling also results in inhibition of NF-кβ activation and has a potentially important anti-inflammatory effect in the vasculature.^38^ Our histology results also showed that IGF-I had a significant role in reducing interstitial edema, leukocyte infiltration, hemorrhage, and myonecrosis. Moreover, Wang et al demonstrated that IGF-I improves diabetic cardiomyopathy as a serious complication of diabetes mellitus via antioxidative and anti-inflammatory mechanisms, as well as with regulation of the Akt/GSK-3 signaling pathway.^39^

It is proclaimed in this study that the effects of combined exercise and IGF-I treatment develop VEGF protein expression and MVD more intensely than NF-кβ and TSP-1 protein expression’s decline through exercise. Also, combination treatment greatly ameliorated cardiac tissue damage and dramatically lowered interstitial edema, leukocyte infiltration, hemorrhage, and myonecrosis compared to the diabetes group. However, the results showed that the influence of exercise in inducing angiogenesis in diabetic heart tissue is greater than insulin. Although, it should be noted that this study still had some defects, such as we used one dose of IGF-I for investigation of its effect on angiogenesis while different doses can be considered. Interestingly, the proangiogenesis role of IGF-I has been proven in several studies. Also, we observed that positive angiogenesis effects could be brought up by exercise and IGF-I and improve heart tissue damages by enhancing VEGF protein level and reducing TSP-1 and NF-кβ proteins expression, but we do not investigate their mechanism of action. Therefore, it is suggested that other studies can be conducted for further findings on larger sample sizes.

## Conclusion

The results conclusively showed that diabetes impairs capillarization and affects the expression of proteins involved in angiogenesis (VEGF-A, TSP-1 and NF-кβ ) in heart tissue. Also, exercise and IGF-I can improve lower angiogenesis levels in the diabetic heart by declining effect on TSP-1 and NF-кβ and increasing effect on VEGF-A proteins expression. Thus, presented outcomes may be useful to improve the impaired angiogenic activities through developing a new strategy in cardiovascular diseases.

## Acknowledgments

This study was supported by Student Research Committee, Urmia University of Medical

Sciences, Urmia, Iran.

## Funding

Funding was provided by Student Research Committee, Urmia University of Medical Sciences ) Contract Number: 10467).

## Ethical approval

The study was approved by the Animal Ethics Committee of the Urmia University of Medical Sciences, (IR.UMSU.REC.1400.242(.

## Competing interest

The authors declare no conflict of interest.
